# Improved access in minimally invasive temporomandibular joint surgery through a novel endaural template

**DOI:** 10.1186/s12893-021-01098-2

**Published:** 2021-02-19

**Authors:** Matthias Krause, Mohammad Kamal, Daniel Kruber, Dirk Halama, Thomas Hierl, Bernd Lethaus, Alexander K. Bartella

**Affiliations:** 1 Department of Oral and Maxillofacial Surgery, Leipzig University, Liebigstraße 12, 04103 Leipzig, Germany; 2 Department of Surgical Sciences, Faculty of Dentistry, Kuwait University, Safat, Kuwait; 3 Faculty of Mechanical and Energy Engineering, University of Applied Sciences (HTWK), Karl-Liebknecht Str. 145, 04277 Leipzig, Germany; 4 Department of Oral and Maxillofacial Surgery, Helios Voigtland-Klinikum Plauen, Röntgenstraße 2, 08529 Plauen, Germany

**Keywords:** TMJ surgery, Template, Endaural template, Minimally invasive temporomandibular joint surgery

## Abstract

**Background:**

Digitally designed surgical templates for minimally invasive temporomandibular joint (TMJ) surgery (MITMJS) are a promising tool for improving the safety of these procedures. Given the TMJ anatomy, the template fitting and intraoperative overview are the most important issues for a safe surgery. This article is a technical advance article that aims to describe an endaural surgical template based on the Moses approach as a possible solution in TMJ surgery.

**Methods:**

Three patients with internal derangement were treated with the guidance of a MITMJS template based on cone beam computed tomography (CBCT) and a surface imprint of the periauricular region. None of the patients needed an additional open surgical procedure. Fitting of the templates was judged in terms of position and rotational stability. Surgical side effects and complications were recorded for each patient.

**Results:**

The template design and clinical use were satisfactory for MITMJS. The templates showed satisfying fit and good visibility. In the study cohort, no bleeding, facial nerve injury, or other complications occurred after the procedure, and no visible scars were noted postoperatively.

**Conclusion:**

Our feasibility report on template-guided MITMJS shows a promising new application of templates. It points to improved access in arthroscopy or arthrocentesis of TMJ surgery through endaural access with an increased level of safety during surgery.

## Background

The advocation of minimally invasive approaches to temporomandibular joint (TMJ) surgery was first published in 1975 [1]. The proposed advantages of such controlled access include lower surgical morbidity, faster surgical recovery, and a decreased chance of complications, such as facial nerve injury, bleeding, perforation of the external acoustic meatus or the articular cavity, as described in previous studies. [1–3] However, proper conduction of minimally invasive treatment requires an advanced level of expertise in the field of minimally invasive temporomandibular joint surgery (MITMJS). To simplify and standardize the approach, we described the use of a computer-aided designed and computer-aided manufactured (CAD-CAM) template for TMJ surgery in 2019, yet several modifications have been proposed to this promising technology. [4] Most of the described modifications require 3D imaging (e.g., CT, cone beam CT, MRI) of the corresponding TMJ and the surrounding tissue, in addition to an optical three-dimensional (3D) scan of the face. Out of these data, a stereolithic template was designed. Given the TMJ anatomy, the fitting and unique placement of the template is of utmost importance to safely conduct the surgery.

To ensure proper fitting of the 3D surgical template, several designs have been proposed. Some surgeons prefer extensions of the template to the zygomatic bone and forehead, while others rely on occlusal and dental stabilization methods. Each design has its own drawbacks, as the design may position the fixation out of the primary field of view of the surgeon, so if the template dislocates slightly, this might go unnoticed, leading to errors. In addition, dental fixation of the template may lead to increased exposure to bacteria of the oral cavity. Another drawback in the currently described templates is that they are designed to be utilized only through the preauricular approach. This risks accidentally perforating the external acoustic meatus. Additionally, visualization and instrumentation of the lateral and medial joint landmarks might be difficult to handle due to anatomical limitations. This is a known surgical problem in arthroscopic TMJ surgery, which was addressed by Moses and Poker in 1989 [5]. They described an enaural approach to the TMJ leading to an increased field view and allowing for improved surgical handling.

In our study, we evaluated the conductibility of TMJ surgery using a CAD/CAM-based template through Moses’ endural approach.

## Materials

### Patients and selection

This study was designed as a technical advance article. It was approved by the Local Ethics Committee at Leipzig University Hospital following the Declaration of Helsinki on medical protocols and ethics (Eth-30/17, 12/06/2017). Three patients of a tertiary care center who needed MITMS were included in the study from March to August 2020. The patients were diagnosed with an internal derangement (ID) of the TMJ, which was classified according to the Wilkes classification system [6]. All patients underwent TMJ surgery using the guided endaural template. None of the patients needed additional open surgical procedures. Follow-up time was three months. Outcome variables were categorical. We noted fitting of the templates, which was judged by the surgeon in terms of position and rotational stability. Furthermore, surgical side effects and complications were recorded for each patient. Patients with effect modifiers or a potential confounder, which would require an additional open approach, were excluded from the study because the template would no longer fit.

### Surgical procedure

**Fig. 1:**
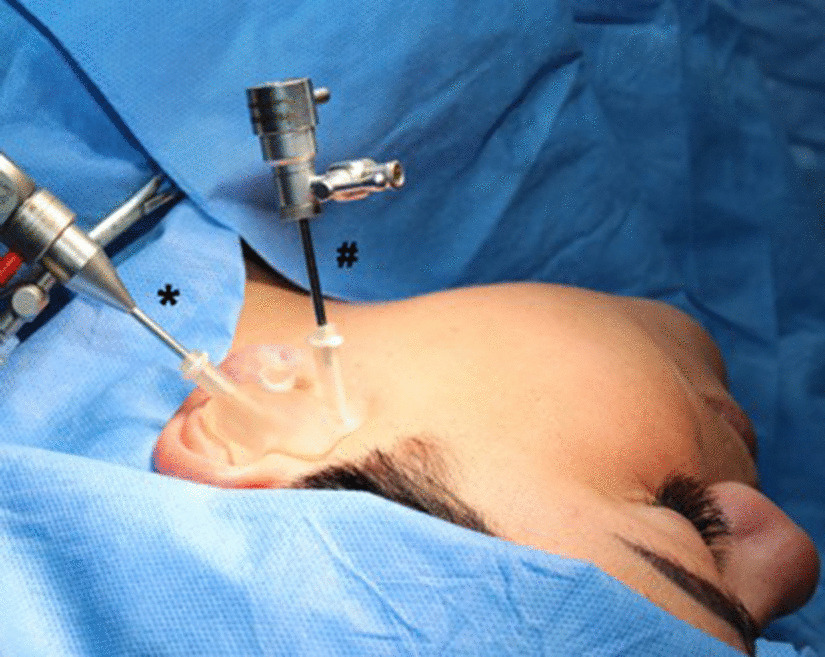
Intraoperative situation after insertion of the printed template for arthroscopy. The endaural approach is marked with an * and the arthroscopic instruments are inserted via the pilot channel. The trocar for cannula or manipulations instruments is inserted via the posterolateral approach (#). The authors recommend a 0° angle for the optic. The central buckle is additional photopolymer/resin and improves the intraoperative handling

The surgical procedure was conducted under sedation or general anesthesia. The template was positioned in the ear, and the skin was marked through the pilot channel for the desired incision after insertion of the trocar. The template was temporarily removed, and the subcutaneous tissue was spread with fine-point scissors. After the guide had been repositioned, while the mandible was distracted downward and forward, the anterior wall of the external auditory canal was perforated with the sharp trocar up to the capsule (endaural access). Next, the endoscope was inserted through the other pilot channel after marking and incising the skin (Fig. 1). The authors preferred a 0° arthroscopic cannula for the procedures.

### Template manufacturing

**Fig. 2:**
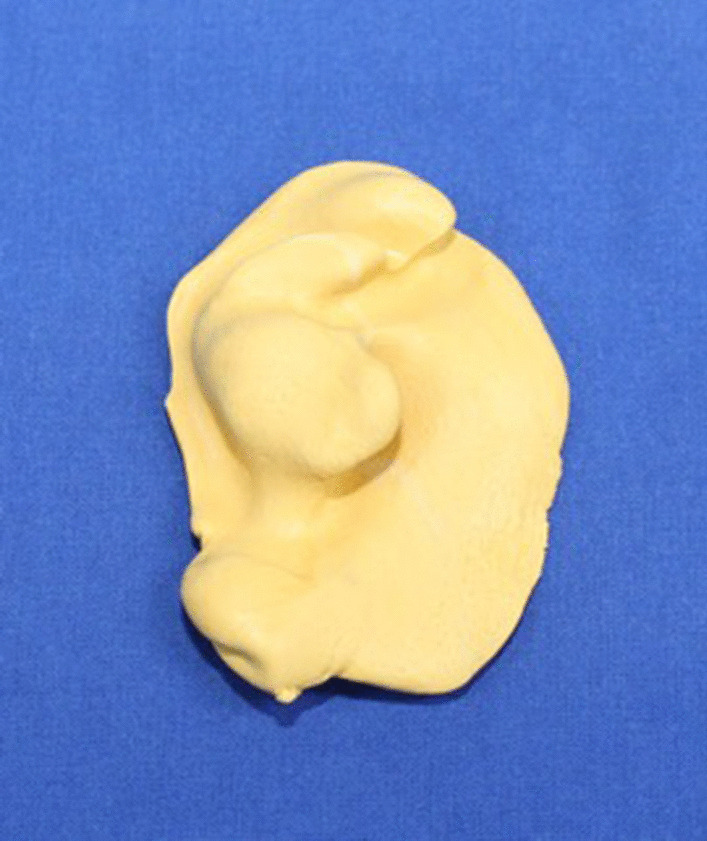
The conventional imprint with polyvinylsiloxane. Detailed capture of the three-dimensional external meatus is possible

**Fig. 3:**
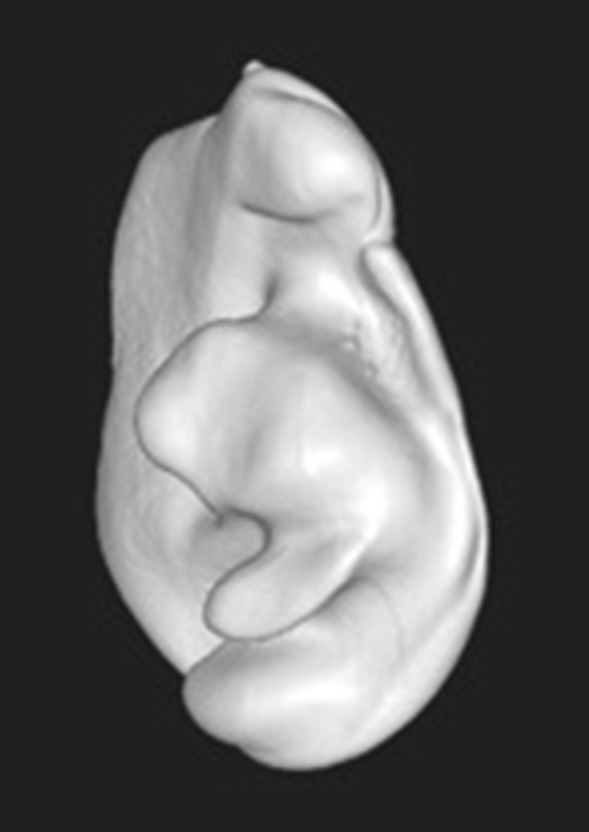
Converting the impression to a virtual three-dimensional data set that can be imported to planning software. In this case, a CBCT-scan was used

**Fig. 4:**
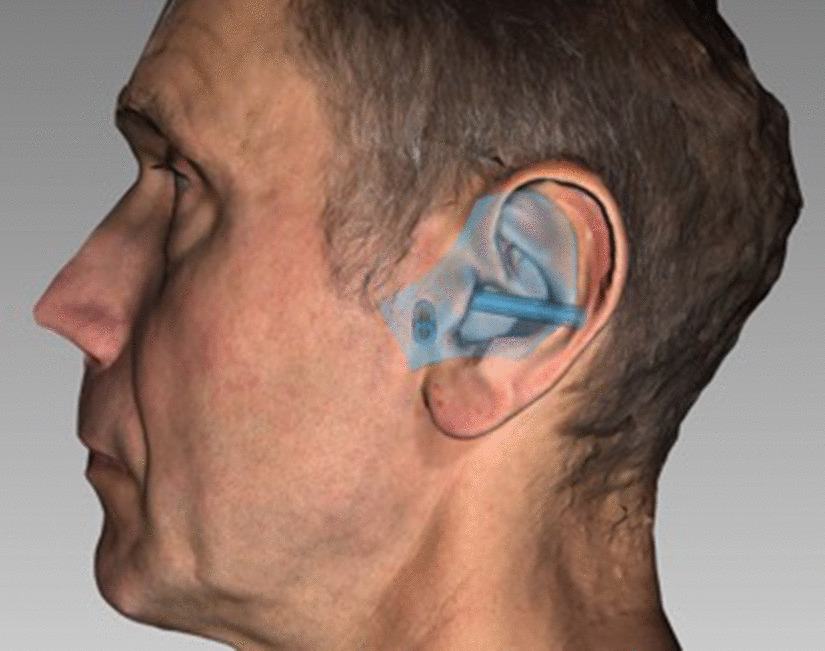
Import of the previously generated 3D data set and design of the template. It was planned in FAT software. The view with skin surface is displayed

**Fig. 5:**
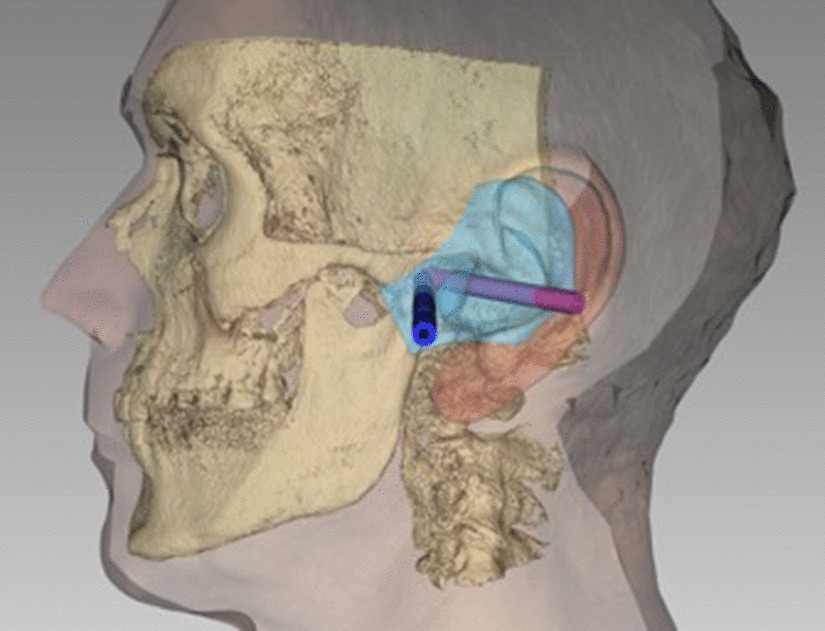
Planning the pilot channels in FAT software. View in bone surface and direction of the pilot channels. They end at the TMJ capsule

**Fig. 6:**
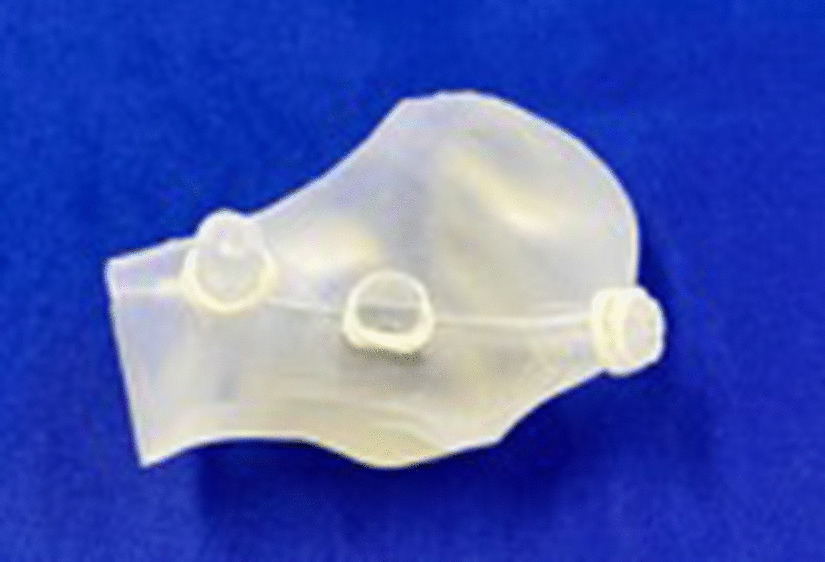
The final template 3D printed. They are composed of biocompatible photopolymer resin

The production of an endaural template was conducted according to the following four steps:

1. A 3D data set of the patient’s skull was created. This was done by computed tomography (CT), cone beam computed tomography (CBCT), or magnetic resonance imaging (MRI). In our cohort, CBCT data sets were used for all patients (Kodak 9500 3D; Carestream Health, Toulouse, France).
2. The surface imprint of the end- and preauricular region was assessed. A precise acquisition was necessary for the template’s position and rotational stability (Fig. 2). The external acoustic meatus was used as a “key lock structure”, and an impression had to be taken using silicone material (Omnisil; Omnident Dental, Rodgau, Germany). The transfer of the imprint to a digital model was performed with CBCT from the plaster model (Fig. 3) due to the availability of the device and ease of image fusion (Fig. 4).
3. The data sets were fused in planning software (Facial Analysis Tool (FAT)). It was then possible to create the template with two pilot channels for the endoscope and the manipulation instruments from the superior-posterolateral and endaural approaches. The alignment was directed to the upper joint space (Fig. 5).
4. Finally, the template was manufactured using a 3D printer (Formlab 2; Formlabs Inc., Somerville, USA) and a CE-certified biocompatible photopolymer resin (Dental SG Resin; Formlabs, Somerville, USA) (Fig. 6). Due to the small number of patients, no statistical analysis was performed.

## Results

The data for all patients was complete. Three patients with internal derangement of three joints were treated with template guidance. The mean age was 51 years. Treatments and procedures are listed in Table 1. One patient was treated with lavage followed by injections of platelet-rich fibrin (PRF). One patient suffered from Wilkes class II, and two suffered from Wilkes class IV (Table 1). No bleeding, facial nerve injury, or other complications occurred after the procedure. The fitting of the template was satisfactory. It was stable in terms of positioning and rotation. The template workflow took an additional 120 min.

**Table 1:** Patient data and performed surgical procedures (n = 3)

Gender	Age	Diagnosis	Wilkes stage	Side treated	Procedure
Female	34	Internal derangement	II	left	Arthroscopy, lavage
Female	67	internal derangement	IV	right	Arthroscopy, lavage
Male	52	internal derangement	IV	left	Arthroscopy, lavage, PRF-injection
Average	51				

The number of patients, sex, age, diagnosis, and procedure are shown

## Discussion

Minimally invasive surgery is widely utilized and sometimes regarded as the gold standard of treatment in several fields of medicine [7–9]. However, it is difficult in TMJ surgery due to the close proximity of anatomical structures. Several approaches are described in the literature for MITMJ surgery. The major drawbacks of the common anterior approaches are potential complications such as facial nerve injury or otic injury [10]. A solution to this problem was suggested by Moses et al. and their endaural approach to the TMJ [5].

Until now, this approach could not be transferred to a template. The major difficulties for transferring the pilot channels appeared to be the insufficient detection of the internal acoustic meatus by the face scanner, which is used in planning, a problem that we also faced in the treatment process. To solve this problem, we used a silicone impression of the external meatus. It has the drawback that the workflow is not entirely digital. However, the impression material can be easily digitalized, which leads to a perfect fit of the template. Until further improvement of 3D scanning is achieved, it remains the only reliable option to capture the anatomical morphological details.

Another drawback of the templates that were described in the literature is the potential for intraoperative dislocation and loss of positional accuracy. Most templates are designed with wings that reach the zygomatic bone or the forehead. However, given the elasticity of the skin and subdermal tissue, intraoperative dislocation might occur. Due to the design of the templates, dislocation might appear without the surgeon being aware of it. Because of the anatomical proximity to neighboring structures, the dislocation potentially leads to accidental nerve or vessel injury or perforations of the temporomandibular fossa or the acoustic meatus. The use of the external acoustic meatus as the locking structure for the template allows reliable and solid fixation. Furthermore, the surgeon has the complete template in their field of vision to detect a potential dislocation.

From the surgical point of view, Moses and Poker’s technique offers a safe and considerably easy approach for treating the mandibular joint. The described advantages, such as facial nerve protection, can be confirmed in template-guided minimally invasive approaches without visible scars.

The bias and limitations of the study are attributed mostly to the small study sample. Furthermore, we believe that a fully digital workflow with a 3D scan of the endaural region might be more favorable to silicone impressions due to the less time needed.

## Conclusion

Our feasibility report on template-guided MITMJS shows its usefulness and ability to handle new applications of templates. It proposes improved and safe access in arthroscopy or arthrocentesis of temporo-mandibular joint surgery through a novel endaural template.

## Data Availability

Data can be provided. All data can be shared.

## Abbreviations

MITMJS: Minimally invasive temporomandibular joint surgery
TMJ: Temporomandibular joint
CBCT: Cone beam computed tomography
CAD-CAM: Computer aided manufactured
3D: Three-dimensional
ID: Internal derangement
MRI: Magnetic resonance imaging
FAT: Facial analysis tool
PRF: Platelet rich fibrin
